# Gene Expression Analysis of Forskolin Treated Basilar Papillae Identifies MicroRNA181a as a Mediator of Proliferation

**DOI:** 10.1371/journal.pone.0011502

**Published:** 2010-07-09

**Authors:** Corey S. Frucht, Mohamed Uduman, Jamie L. Duke, Steven H. Kleinstein, Joseph Santos-Sacchi, Dhasakumar S. Navaratnam

**Affiliations:** 1 Medical Scientist Training Program, Yale School of Medicine, New Haven, Connecticut, United States of America; 2 Interdepartmental Neuroscience Program, Yale University, New Haven, Connecticut, United States of America; 3 Interdepartmental Program in Computation Biology and Bioinformatics, Yale University, New Haven, Connecticut, United States of America; 4 Department of Pathology, Yale School of Medicine, New Haven, Connecticut, United States of America; 5 Division of Otolaryngology, Department of Surgery, Yale School of Medicine, New Haven, Connecticut, United States of America; 6 Department of Cellular and Molecular Physiology, Yale School of Medicine, New Haven, Connecticut, United States of America; 7 Department of Neurobiology, Yale School of Medicine, New Haven, Connecticut, United States of America; 8 Department of Neurology, Yale School of Medicine, New Haven, Connecticut, United States of America; King Abdullah University of Science and Technology, Saudi Arabia

## Abstract

**Background:**

Auditory hair cells spontaneously regenerate following injury in birds but not mammals. A better understanding of the molecular events underlying hair cell regeneration in birds may allow for identification and eventually manipulation of relevant pathways in mammals to stimulate regeneration and restore hearing in deaf patients.

**Methodology/Principal Findings:**

Gene expression was profiled in forskolin treated (i.e., proliferating) and quiescent control auditory epithelia of post-hatch chicks using an Affymetrix whole-genome chicken array after 24 (n = 6), 48 (n = 6), and 72 (n = 12) hours in culture. In the forskolin-treated epithelia there was significant (p<0.05; >two-fold change) upregulation of many genes thought to be relevant to cell cycle control and inner ear development. Gene set enrichment analysis was performed on the data and identified myriad microRNAs that are likely to be upregulated in the regenerating tissue, including microRNA181a (miR181a), which is known to mediate proliferation in other systems. Functional experiments showed that miR181a overexpression is sufficient to stimulate proliferation within the basilar papilla, as assayed by BrdU incorporation. Further, some of the newly produced cells express the early hair cell marker myosin VI, suggesting that miR181a transfection can result in the production of new hair cells.

**Conclusions/Significance:**

These studies have identified a single microRNA, miR181a, that can cause proliferation in the chicken auditory epithelium with production of new hair cells.

## Introduction

Sensorineural hearing loss represents a major public health concern. Approximately 300 million people worldwide have moderate to profound hearing loss in both ears [1]. Loss of inner ear hair cells, which serve to transduce sound into neural impulses, is responsible for the majority of hearing loss. In humans, and other mammals, loss of hair cells is permanent since these organisms have no capacity for hair cell regeneration. In contrast, other non-mammalian vertebrates such as birds, reptiles, amphibian and fish are able to replace lost hair cells.

The basilar papilla, the avian auditory epithelium, is able to regenerate hair cells in response to hair cell loss (reviewed in [2]). However, the auditory epithelium shows no mitotic activity normally, a feature that is reminiscent of the mammalian auditory epithelium, and contrasts with the chick vestibular epithelium which shows continuous mitotic activity [3]. Thus, the avian auditory epithelium can be viewed as an intermediary in the evolution from the fish to the mammal, and we reason that the study of this epithelium will provide insight into why the mammalian auditory epithelium shows mitotic quiescence at rest (similar to the avian auditory epithelium), but is unable to proliferate in response to damage (in contrast to the avian auditory epithelium).

The basilar papilla is comprised of both sensory transducing hair cells and supporting cells. Following injury, it is the supporting cells which give rise to new hair cells [4], [5]. For example, exposure of birds to intense noise causes some supporting cells to leave growth-arrest, re-enter the cell cycle and ultimately differentiate into hair cells [6], [7], [8], [9]. New hair cells are first seen 4–5 days after the onset of exposure to an intense sound [6], [10] and undergo maturation so that by 20–28 days after stimulus onset they are virtually indistinguishable from unaffected cells [11]. In addition, some new hair cells arise from direct differentiation of supporting cells without an intervening mitotic step [12], [13], [14], [15], [16], [17], [18], [19]. After acoustic or ototoxic insult, birds initially have increased hearing thresholds, which eventually return nearly to baseline confirming that newly produced hair cells are functional [20]. It is believed that this recovery of function results from both regeneration of new hair cells as well as repair of those that have survived [21].

Though the intracellular pathways required for hair cell regeneration have not yet been fully elucidated, various pathways and signaling cascades have been implicated in this process. For example, it has been shown that treatment of the chick basilar papilla with forskolin, a potent adenylate cyclase activator that increases intracellular cAMP levels, causes a robust and widespread proliferation of supporting cells, leading to the production of new hair cells [22]. This effect is first seen after 72 hours in culture, occurs without upregulation of markers of apoptosis, and is significantly blocked by protein kinase A inhibitors. It therefore appears as though activation of this pathway can stimulate growth of new hair cells with limited cell injury. Forskolin also appears to have a mitogenic effect in the mammalian vestibular system, in which brief treatment with this compound results in an increase an supporting cell S-phase entry [23]. Moreover, the same study found that this effect is blocked by brief treatment with forskolin in the presence of monensin or bafilmycin, which inhibit recycling of membrane receptors. Increased cAMP levels may therefore lead to proliferation by causing upregulation of tyrosine kinase growth factor receptors. With age-related hearing loss in humans there is a substantial loss of hair cells within the organ of Corti [24], so it is likely that a robust proliferative process that produces new hair cells is what will ultimately lead to maximal recovery of function in hearing impaired patients. In the present study, microarrays were used to analyze gene expression in forskolin treated basilar papillae given the robustness of this effect.

In other systems, microarray analysis of differing phenotypes has revealed complex differences in mRNA expression. These complex differences are likely brought about by the combined activity of transcription factors and microRNAs (miRNAs) that regulate expression of multiple genes and specific genetic programs. In fact, a study of transcription factor expression in regenerating versus non-regenerating chicken basilar papillae revealed a number of transcription factors that were differentially expressed [25].

MiRNAs are a species of small regulatory RNA that bind to complementary sequences in mRNA and decrease gene expression by increasing degradation and blocking translation (reviewed in [26]). Though each miRNA binds a specific sequence, these molecules are promiscuous in that their complementary sequences are found in a number of different mRNAs [27]. As a result, miRNA expression may represent a way in which cells can rapidly and in a coordinated fashion up- or downregulate a large number of genes. Some miRNAs are known to be important for inner ear development [28], [29], [30], [31] and may play a role in hair cell regeneration, where there is presumably some reiteration of developmental events. However there have been few published studies on the role of miRNAs in auditory hair cell regeneration, specifically. One study revealed that the *let-7* family of miRNA are downregulated in the newt inner ear after hair cell injury [32]. This particular study underscores the importance of examining more closely the role of miRNA in hair cell regeneration.

In order to gain insight into the molecular events which underlie auditory hair cell regeneration in chicken, we compared gene expression in forskolin treated (i.e., proliferating) and control (i.e., quiescent) auditory epithelia using Affymetrix whole genome chicken microarrays. The microarray data were validated by quantitative PCR. Gene set enrichment analyses were performed to identify miRNAs and transcription factors that may be important for hair cell regeneration. Many sets of genes representing predicted targets of specific miRNAs were found to be significantly enriched among genes downregulated in the forskolin-treated sensory epithelia after 72 hours, suggesting that these miRNAs were likely upregulated in the proliferating tissue. One of these microRNAs, miR181a, was selected for functional experiments given its role in proliferation and suppression of the cell cycle inhibitor p27 in human myeloid leukemia cells [33]. P27 is thought to represent a key barrier to hair cell regeneration in the mammalian inner ear [34], [35]. Basilar papillae transfected with a miR181a precursor had increased numbers of BrdU positive cells than epithelia that were transfected with a non-targeting miRNA. Some cells labeling for BrdU also expressed the early hair cell marker myosin VI, indicating that overexpression of miR181a is capable of producing proliferation with production of new hair cells.

## Materials and Methods

### Basilar papilla explant cultures

Animals were treated in accordance with policies set forth by the Yale Institutional Animal Care and Use Committee (protocol number 2007-10439). Under sterile conditions, cochlear ducts containing the basilar papillae were carefully dissected out of 0-day-old chicks and then individually cultured in DMEM with 10% fetal bovine serum with or without forskolin (final concentration 100 µM, delivered in 1% DMSO) for either 24, 48, or 72 hours at 37°C with 5% CO_2_. Control samples received DMSO at 1% as a vehicle control. At the end of 72 hours, the tegmentum vasculosum was dissected off to expose the auditory epithelium, which was delicately freed from the underlying cartilaginous plates. All explants were kept in culture for 72 hours because new hair cells are first seen in basilar papillae treated with forskolin after ∼72 hours of exposure to the small molecule [22]. Therefore, at even earlier time points in culture the molecular events that underlie hair cell proliferation are well underway. Each sample was comprised of three auditory epithelia from three different chicks and were put in 100 µL of DMEM and then immediately frozen at −80°C until RNA isolation could be performed. There were a total of 24 samples in this experiment: three 24-hour forskolin, three 24-hour control, three 48-hour forskolin, three 48-hour control, six 72-hour forskolin and six 72-hour control.

### RNA isolation

Immediately upon removal of the frozen epithelia samples from the −80°C freezer, the lysis buffer from the RNAqueous® Kit (Ambion, Austin, TX) was added. Given the small amount of tissue and relatively large surface area to size ratio, no measures to actively disrupt or homogenize the tissue in each sample were necessary. Total RNA isolation then proceeded as directed by the RNAqueous® Kit manual. Sample quality was confirmed by gel analysis.

### Microarray experiments

Double-stranded cDNA and biotin-labeled cRNA were synthesized from 1 to 5 µg of total RNA using a two-cycle target labeling kit [36]. Biotin-labeled cRNA was purified using the GeneChip Cleanup Module prior to fragmenting to a size of 35–200 bases by incubating at 94°C for 35 minutes in fragmentation buffer (40 mM Tris-acetate, pH 8.1, 100 mM potassium acetate, 30 mM magnesium acetate). Hybridization of the samples with Affymetrix chicken arrays was then performed at the Yale University Keck Facility according to the manufacturer's protocol [37]. All microarray data is MIAME compliant and has been uploaded to MIAMExpress (ArrayExpress accession: E-MEXP-2642, username = Reviewer_E-MEXP-2642, password = 1269882335242).

### Differential gene expression analysis

The microarray gene expression analysis was performed using GeneSpring GX 9.0 software (Agilent Technologies, Santa Clara, CA). The data were GC-RMA normalized without baseline transformation, and then filtered by expression (20%–100% in at least one of the six or twelve samples at each time point) to exclude genes expressed only at very low levels in all samples. Median expression values were used for redundant probe sets. Genes differentially expressed were identified by using a fold-change cutoff of 2 and then performing an unpaired t-test after Benjamini-Hotchberg correction for multiple comparisons (p<0.05). Heat maps were generated using R version 2.10.1 (www.r-project.org) using the HeatPlus add-on in the Bioconductor package.

### Quantitative PCR validation of microarray gene expression data

In order to validate the microarray data, twelve genes were selected to be verified by qPCR. Seven of the genes selected were upregulated and five were downregulated in the forskolin condition. The upregulated genes validated by qPCR were highly expressed in at least one condition and chosen to span a range in fold-change differences between the two experimental conditions at 72 hours, from 2.0 to 64.3. The 12 genes that were validated are BECN1, CCNI, CDKN1B (p27), CDKN2B, DPM1, EME1, FBXO8, ITGA4, NLGN1, OCM, PIGW, SNX1. Additionally, expression of the 18S ribosomal subunit was also assessed to allow for normalization of total RNA levels between samples using the 2^−ΔΔC^_T_ method [38]. Primers were designed using MacVector (MacVector, Cary, NC) software and Primer 3 [39] and were validated by melting curve analysis. 5 µg of RNA was isolated from each sample as described above and was used to create first-strand cDNA using random hexamers and reverse transcriptase (Superscript II; Invitrogen, Carslbad, CA), according to the manufacturer's instructions. Amplification was performed using the SYBR Green Supermix kit (Bio-Rad, Hercules, CA) on an iCycler system (Bio-Rad, Hercules, CA). Three replicates were performed and the data averaged for each cDNA sample and primer pair combination. Those fold-changes with a 95% confidence interval excluding one were considered significant.

### Gene sets used for enrichment analyses

Gene set enrichment analysis (GSEA) was performed to analyze the pattern of differential gene expression between forskolin treated and control basilar papillae using GSEA version 2 software [40]. Three different gene set packages were used, two of which were downloaded directly from the Broad institute website (www.broad.mit.edu/gsea).

To identify transcription factors which may be important for hair cell regeneration, a GSEA was performed using a gene set package called “c3.all.v2.5.symbols,” which was obtained from the BROAD Institute website. This package contains a series of gene sets defined by the presence of transcription factor motifs and predicted miRNA binding sites. The genes in each set of this package therefore share a *cis*-regulatory motif that is conserved across human, mouse, rat, and dog genomes and will be referred to hereafter as the “mammalian gene sets.” The motifs used come from Xie et al. (2005) and the TRANSFAC database and include sets of genes sharing a 3′-UTR miRNA binding motif [41]. Even though these gene sets are not based on the chicken genome, we reasoned that these results would be of interest given the known conservation of miRNA targets among mammals [42]. We further surmised that if the conservation of miRNA targets is poorly conserved between mammals and non-mammalian vertebrates then an enrichment analysis might produce no statistically significant results. Any significant findings, however, would warrant further examination.

In order to validate the GSEA results from the analyses performed using the mammalian gene sets, GSEA was also run using a gene set package that was generated by scanning the portions of the chicken genome that are conserved with frog (*Xenopus tropicalis*) to identify gene sets sharing particular transcription factor motifs defined in the TRANSFAC database. These gene sets will hereafter be referred to as the “chicken/frog conservation gene sets.” Only conserved regions were used to reduce the number of false positives, as there is evidence to suggest that transcription factor recognition sites tend to be enriched in conserved portions of the genome [43]. However, recent genome-wide studies have demonstrated that many transcription factor binding sites are not conserved [44], [45] and are also found outside of the proximal promoter region [44], [46]. Thus, our approach should be considered to be conservative in the sense that some true binding sites may be overlooked, but those that are identified have a higher likelihood of being functional. The frog genome in particular was used in light of this organism's close evolutionary relationship to chickens. The use of conservation between more distantly related organisms, such as chicken and mammals, would be very strict and would result in the omission of many potentially significant positive results. To create these gene sets we downloaded the transcription start site for all chicken RefSeq genes, defined by the May 2008 RefGene table [47] using the UCSC Genome Bioinformatics site (genome.ucsc.edu). The region 2 kilobases around each transcription start site was identified within the May 2006 genome-wide multiple alignment of 6 vertebrate species to the chicken genome [48], also available through UCSC Genome Bioinformatics site. In order to identify putative transcription factor binding sites, the chicken sequences, along with aligned regions from frog, were analyzed using the TRANSFAC MATCH algorithm with a cutoff chosen to minimize the sum of false positives and false negatives [49]. The analysis was performed for all vertebrate transcription factor matrices in the 2009.1 release of TRANSFAC [50], and putative binding sites were considered to be evolutionarily conserved if matches were also found at the aligned positions in both chicken and frog sequences and had no gaps present in the multiple alignment of these species.

To assess the functional relevance of the pattern of differentially expressed genes across a variety of organisms and systems, GSEA was performed using a gene set package called “c2.all.v2.5.symbols” which was also downloaded from the BROAD Institute website. This package defines curated sets of genes based on specific experimental findings from experiments on human and model animal tissue, which includes canonical pathways and chemical/genetic perturbations based gene sets. These gene sets will hereafter be referred to as the “curated gene sets.” The relevant reference is given on the BROAD website for each curated gene set for assessment of relevance.

### Gene set enrichment analysis

The GeneSpring processed data from the 38,535 original probes was collapsed into 13,159 genes based on gene symbols. Some genes were assigned to one or multiple gene sets downloaded with the GSEA package. There are a total of 837 gene sets in the mammalian gene sets package, 50 of which were excluded by gene set size criteria (15–50), leaving 783 to be included in our analysis. 359 of the 566 chicken/frog conservation gene sets met this size threshold criterion, as did 1,197 out of the 1,892 curated gene sets (Table 1).

**Table 1:** Gene set enrichment analysis results.

Gene Set Package	Statistic Description	24H FSK	24H CTL	48H FSK	48H CTL	72H FSK	72H CTL
Mammalian	Up/Total	407/783	376/783	512/783	271/783	146/783	636/783
	*p*<0.05	72	26	165	29	37	210
	*q*<0.25	66	0	198	5	41	331
Conservation	Up/Total	323/359	36/359	256/359	103/359	219/359	140/359
	*p*<0.05	176	2	127	22	95	31
	*q*<0.25	230	0	162	53	128	45
Curated	Up/Total	691/1,197	507/1,197	778/1,197	419/1,197	695/1,197	502/1,197
	*p*<0.05	240	136	364	94	231	216
	*q*<0.25	323	152	495	114	285	346

The number of mammalian miRNA/transcription factor motif based (“Mammalian”), chicken/frog conservation transcription factor motif based gene sets (“Conservation”), and curated gene sets from the BROAD Institute (“Curated”) meeting the criteria *p*<0.05 and *q* (false discovery rate) <0.25 for forskolin (FSK) and control (CTL) samples after 24, 48, or 72 hours in culture.

Genes were first ranked by their signal-to-noise ratio between the forskolin and control groups. In other words, the genes most consistently overexpressed in the forskolin samples were ranked at one end of the list and those most underexpressed in this group were ranked at the other end. GSEA asks whether the genes that make up a particular gene set are randomly distributed throughout the ranked list of genes, or whether they have a tendency to cluster at the top or bottom of the list. This question was answered by calculating for every gene set a running sum statistic, the maximum of which is referred to as the enrichment score (ES). Each ES is then normalized to the size of its corresponding gene set to produce a normalized enrichment score (NES). Statistical significance of each NES is determined by comparing that NES to the distribution of ESs generated by randomly permutating the genotype class labels of the data set. Therefore, the null hypothesis of a GSEA is that the distribution of a gene set throughout the ranked list of genes is random with regards to the two treatment conditions being compared. The alternative hypothesis is that location of genes in a set is associated with the treatment conditions. Both the p-value and false discovery rate (FDR; q-value) were calculated for each set. Gene sets with a FDR less than 0.25 and a p-value less than 0.05 were considered differentially expressed. The GSEA parameters used were as follows: metric = signal to noise; permutation number = 1,000; gene size minimum = 15; gene size maximum = 500; enrichment statistic = classic; permutation type = phenotype. One GSEA was performed using the 24-hour control data and 72-hour control data to negatively control for gene expression changes occurring with culture duration, as well as false positives not reflective of the treatment condition.

### Pre-microRNA181a and anti-microRNA181a transfection

For functional miR181a experiments, basilar papilla explants from 0-day-old chicks were cultured with or without forskolin for 72 hours as described above. Basilar papillae from both conditions were also transfected with either a miR181a precursor (Ambion, Austin, TX) or a non-targeting miRNA which served as a negative control (Ambion, Austin, TX) at a final concentration of 100 nM. Transfections were performed for six hours in serum-free medium which was eventually replaced with regular medium. BrdU was present at a concentration of 0.01% for the entire culture duration, except for the six-hour transfection. Some basilar papillae were also cultured for 24 hours with forskolin as described above and then transfected with anti-miR181a (Ambion, Austin, TX) to suppress endogenous miR181a. Transfections were performed using the lipid-based X-tremeGENE SiRNA Transfection Reagent (Roche, Indianapolis, IN) per the manufacturer's instructions.

### Antibody labeling

After 72 hours in culture basilar papillae were fixed in 4% PFA in PBS for 30 minutes. All fixative was removed by three 5-minute washes in PBS. The tissue was blocked and permeabilized using a solution of PBS with FBS (10%) and Triton-X (0.1%) for one hour. Each basilar papilla was then incubated with mouse anti-BrdU antibodies (1∶40, BD, Franklin Lakes, NJ) in PBS for one hour. After washing with PBS, alkaline phosphatase-conjugated goat anti-mouse IgG (1∶400, Santa Cruz, Santa Cruz, CA) in PBS with Triton-X (0.1%) was added for one hour. After washing in PBS, alkaline phosphatase substrate was produced using the NBT/BCIP Reagent Kit (Invitrogen, Carlsbad, CA) per the manufacturer's instructions and then added to the tissue for approximately five minutes. All substrate was then thoroughly washed with PBS.

### Statistics

Basilar papillae immunohistochemically labeled for BrdU were viewed using brightfield microscopy and were imaged using a digital camera attached to a Zeiss Stemi SV 11 stereoscope. The borders of the sensory epithelium were easily viewed both by direct microscopy and in the captured images. The intraepithelial location of BrdU positive nuclei was confirmed through focal plane adjustments. Only those nuclei which were clearly within the epithelium were counted. Unpaired Student's t-tests were performed to determine the statistical significance of the comparisons discussed in the text using a cut-off of p<0.05.

### Co-labeling experiments

To determine whether any newly produced BrdU positive cells express the early hair cell marker myosin VI, some basilar papillae were transfected with pre-miR181a as described above and cultured for 72 hours. At the end of the culture duration, the tegmentum vasculosum and tectorial membranes were removed prior to fixation in 4% PFA for 30 minutes. Basilar papillae were cryoprotected overnight in 30% sucrose and then embedded in PBS with 1% low melting point agarose and 18% sucrose. The tissue was cryosectioned at 10 µm and then blocked and permeabilized as described above. Sections were labeled for both myosin VI and BrdU as previously described [12]. Sections were then incubated with rabbit anti-myosin VI (1∶350, Proteus, Ramona, CA) in blocking solution for one hour. After washing, sections were then incubated in Alexa Fluor 546 conjugated anti-rabbit secondary antibodies (1∶1,000, Invitrogen, Carlsbad, CA) for one hour. After washing, basilar papilla sections were post-fixed in 4% PFA for one hour. The cryosections were then washed in PBS before 20 minute 2M HCl treatment to expose BrdU. After thorough washing, sections were incubated with mouse anti-BrdU antibodies (1∶40, BD, Franklin Lakes, NJ) for one hour. After 3 additional PBS washes, sections were incubated with Alexa Fluor 647 conjugated anti-mouse secondary antibodies (Cell Signaling Technology, Danvers, MA) for one hour. After thorough washing, sections were mounted on glass slides in Vectashield fluorescent mounting medium (Vector Laboratories, Burlingame, CA). Images were captured using a Zeiss LSM 510 confocal microscope.

## Results and Discussion

### Differential expression of genes in the forskolin and control groups increases as a function of time

We report here a systematic comparison of genome-wide expression between proliferating and non-proliferating chick auditory epithelia using Affymetrix whole genome chicken microarrays. The forskolin-induced proliferation paradigm was chosen for this study given the robustness of the effect, which we reasoned would facilitate identification of the molecular events resulting in the production of new hair cells. RNA was isolated from control and forskolin-treated samples cultured for 24, 48 and 72 hours. BrdU incorporation, a marker for S-phase entry, is seen at 72 hours in the forskolin paradigm. Since S-phase entry is the criterion that distinguishes the avian epithelium from that in mammals, we have concentrated our analysis on this time point.

**Figure 1:**
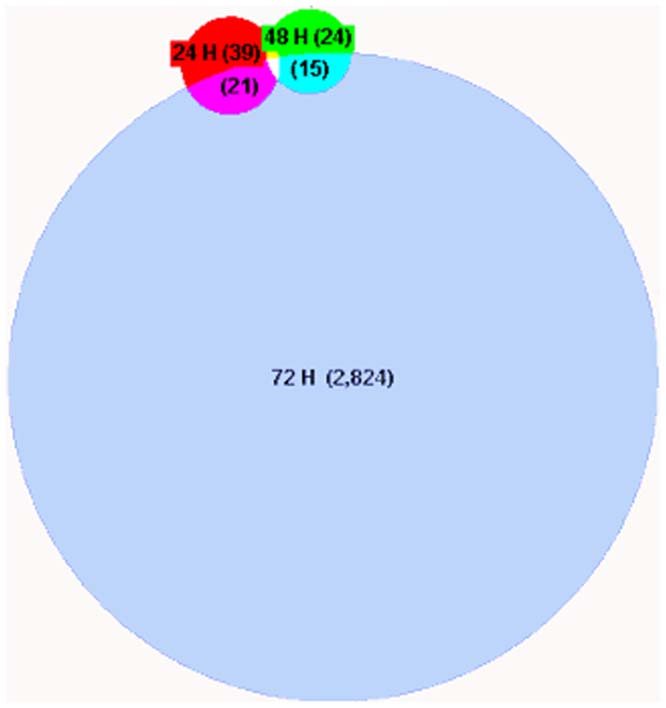
Far more genes were differentially expressed at 72 hours than at 24 or 48 hours of exposure to forskolin. Shown is a proportional Venn diagram indicating the number of genes significantly (p<0.05 and fold change >2) differentially expressed at 24, 48, and 72 hours of exposure to forskolin. The number of genes in each category is indicated in parentheses. Far more genes are affected at 72 hours than at either of the two earlier time points. A little over half of the genes differentially expressed at 24 or 48 hours are also differentially expressed at 72 hours. Only one gene, AMPH, was affected at all three time points.

**Figure 2:**
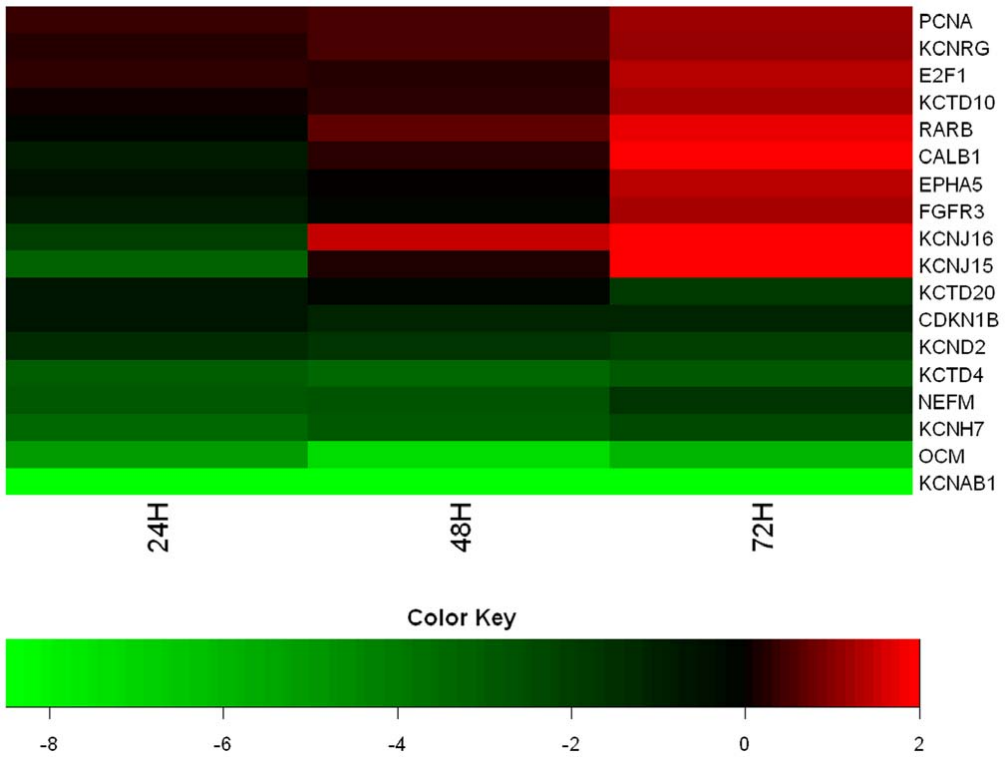
Fold-change differences of selected genes of interest remain stable at 24, 48, and 72 hours. Shown is a heat map depicting fold-changes between the forskolin and control conditions for a set of genes of interest the three time points indicated. The genes shown were selected because of their known expression in the inner ear (i.e., RARB, CALB1, OCM), ion channel identity (e.g., KCNAB1, KCNJ15), or relevance to the present study (i.e., E2F1). The genes span a range of fold-changes and are both up and downregulated with forskolin treatment. Fold-changes are given as log_2_(expression in forskolin/expression in control). The directionality of fold-changes was fairly consistent across time points, but there was a slight general trend toward increasing fold-change values from 24 to 72 hours.

The isolated RNA from proliferating and non-proliferating auditory epithelia was amplified, biotinylated, hybridized to Affymetrix chicken arrays and scanned as described. The normalized data were then filtered by expression to exclude all probes that were expressed below 20% of the maximum expression level in at least one of the samples at each time point. This measure was taken to ensure that differential expression of genes expressed at very low levels were not assigned a disproportionate amount of significance. 32,509 out of 38,535 probes in the microarray met this initial criterion in the 72-hour samples (Table 2). Of the 32,509 filtered probes, 5,032 had a corrected p-value less than 0.05 at 72 hours. Of these 5,032 genes, 2,824 had at least a two-fold change in expression, which was used as a criterion for functional significance. The same statistics from the 24- and 48-hour samples are also shown in Table 2. A proportional Venn diagram clearly shows differential expression of far more genes at 72 hours than at 24 or 48 hours, but expression of most of the genes differentially expressed at the two earlier time points are also affected at 72 hours (Figure 1). It is not surprising that far fewer genes are differentially expressed at 24 and 48 hours than at 72 hours, given that there must be some latency following exposure to forskolin before gene expression is affected. A heat map revealed that directionality of fold-changes for specific genes remained fairly consistent at 24, 48, and 72 hours (Figure 2). There also appeared to be a general trend toward larger fold-change differences at the two later time points.

**Table 2:** Microarray analysis statistics.

	24 H			48 H			72 H		
Statistical Filtering	Number of Probe Sets	Probe Sets F>C	Probe Sets F<C	Number of Probe Sets	Probe Sets F>C	Probe Sets F<C	Number of Probe Sets	Probe Sets F>C	Probe Sets F<C
Total	38,535			38,535			38,535		
Expression above background^a^	31,692			31,793			32,509		
Corrected *p*<0.05	46			25			5,032		
>1.5-fold (of p<0.05 list)	41	9	32	25	7	18	4,313	2,236	2,077
**>2-fold (of ** ***p*** **<0.05 list)**	**39**	**9**	**30**	**24**	**6**	**18**	**2,824**	**1,291**	**1,533**
>3-fold (of p<0.05 list)	28	3	25	20	2	18	1,302	432	870
>4-fold (of p<0.05 list)	26	3	23	18	1	17	760	194	566

*^a^*The entire gene expression data set was filtered to exclude probes whose expression was not at least 20% in at least one out of the six 24-hour samples (**24 H**), six 48-hour samples (**48 H**), or twelve 72-hour samples (**72 H**). The statistics reported are based only on those probes that met this criterion. Half of the samples from each time point were treated with forskolin (F) and the remainder served as controls (C). Showed in boldface are the statistics for the list of genes defined to be differentially expressed using p<0.05 and fold-change >2.

Many genes thought to be related to cellular proliferation and inner ear development were differentially expressed between the two conditions at multiple time points (Table 3). The complete lists of genes differentially expressed between the control and forskolin conditions at 24, 48, and 72 hours are included as Supporting Information (Tables S1, S2, and S3, respectively). Given that cellular proliferation is first observed in the basilar papilla at 72 hours in culture with forskolin, the following discussion will focus on data from this particular time point. One gene that is differentially expressed after 72 hours of exposure to forskolin is cyclin I (CCNI), a cell cycle regulator, which is over three-fold underexpressed in the forskolin samples at 72 hours and is downregulated in proliferating murine cardiomyocytes (Table 3) [51]. Wnt5a is underexpressed in regenerating tissue, a noteworthy observation as inactivation of this gene has been linked to cellular proliferation in a particular form of leukemia [52]. Wnt proteins are also thought to play a role in the development of the lateral line in zebrafish (reviewed in [53]) and chicken inner ears [54], [55], [56]. Frizzled homolog 10, a gene likely to play a role in cellular proliferation, is over 15-fold overexpressed in proliferating versus non-proliferating tissue after 72 hours in culture [57].

**Table 3:** QPCR validation of gene expression data.

	Microarray24 Hours		Microarray48 Hours		Microarray72 Hours		qPCR72 Hours
*Gene symbol*	Median Fold-Change(F/C)	Corrected *p* Value	Median Fold-Change(F/C)	Corrected *p* Value	Median Fold-Change(F/C)	Corrected *p* Value	Average Fold-Change(F/C)
CCNI	1/1.68	0.26	1/2.46	0.28	1/3.00	5.22E-05	1/1.54
	1/2.24	0.27	1/4.59	0.25	1/3.31	0.0017	
	1/2.57	0.23	1/3.32	0.33	1/3.44	0.0035	
	1/2.27	0.23	1/2.90	0.34	1/4.21	3.63E-04	
WNT5a	1/3.88	0.15	1/2.56	0.41	1/2.31	0.017	
WNT3	1.26	0.40	1.90	0.41	2.81	.023	
FZD10	1.28	0.86	1/1.34	0.88	15.48	0.0092	
BECN1	1.05	0.88	1.65	0.37	2.04	0.051	1.66
	1.39	0.27	1.37	0.50	1.61	0.15	
CDC45L	3.15	0.34	6.19	0.51	3.16	0.14	
DPM1	1.10	0.79	1.51	0.19	2.40	5.00E-04	1/1.11
	1.17	0.52	1.47	0.51	2.15	0.0024	
SNX1	1.69	0.21	1.22	0.39	2.65	7.07E-04	5.62
EME1	1.03	0.27	1.03	0.65	5.29	0.0032	1.38
	1.99	0.25	3.52	0.36	1.08	0.31	
FBXO8	1.03	0.27	2.02	0.22	3.42	0.0072	1.96
	1/1.99	0.25	2.05	0.37	2.16	0.031	
PIGW	2.09	0.041	2.58	0.33	4.30	6.38E-04	1.64
CDH20	1/1.11	0.87	1/1.28	0.56	1/8.20	0.0018	1/8.11
	1.13	0.89	1/2.19	0.51	1/22.25	4.52E-05	
ITGA4	1.09	0.36	1/1.18	0.51	1/1.06	0.47	1/3.67
	1/1.87	0.080	1/1.40	0.49	1/2.08	0.089	
	1.16	0.47	1/1.42	0.51	1/2.28	0.055	
NLGN1	1.06	0.30	1/1.12	0.51	1.02	0.43	1/1.46
	1.08	0.27	1/1.08	0.51	1/1.01	0.87	
	1/5.92	0.23	1/3.90	0.15	1/1.25	0.45	
	1/2.58	0.27	1/3.08	0.51	1/2.11	0.0046	
	1.06	0.28	1/1.05	0.51	1/2.99	0.011	
CDKN1B (p27)	1/1.27	0.30	1/2.97	0.49	1/2.12	0.015	2.64
	1/1.77	0.15	1/1.72	0.22	1/2.21	9.92E-04	
OCM	1/1.27	0.307	1/153.66	0.14	1/64.33	1.81E-04	1/13.45

QPCR and microarray data for individual genes of interest and genes selected for qPCR validation as described in the text. Multiple microarray values represent different probe sets for the same gene.

### Differential gene expression is confirmed by qPCR

Quantitative PCR (qPCR) was performed on select genes in order to validate the microarray data. Primers were designed for twelve genes that were found by microarray to be differentially expressed between the two conditions at 72 hours; seven genes were upregulated and five were downregulated in the forskolin condition. QPCR confirmed the directionality of the microarray findings for nine out of the twelve genes tested (75%). The fold-changes of these nine genes were all significantly different from one (95% confidence interval, data not shown), confirming their differential expression as detected by microarray. These findings validate the initial microarray results, so further analyses were performed on the gene expression data set.

### Curated gene set enrichment results

Our initial analysis of expression of individual genes that were differentially expressed revealed that many are known to be involved in control of the cell cycle and hair cell differentiation. To more systematically identify genes that were correlated with specific phenotypes, we used gene set enrichment analysis (GSEA), a computational method to determine the expression of genes associated with specific empirically determined phenotypes. Gene sets packages were downloaded from the Broad Institute website and were used for GSEAs on the Affymetrix expression data from 24, 48, and 72 hours (Table 1). An additional GSEA was performed comparing the microarray data from the 24-hour control samples to that in the 72-hour control samples to serve as a negative control for changes in gene expression resulting from the culture conditions rather than exposure to forskolin. This GSEA produced no significantly (FDR <0.25 and p-value <0.05) enriched gene sets for any of the three gene set packages used (data not shown).

To assess the functional relevance of the genes that were differentially expressed, a GSEA was performed using manually curated gene sets downloaded from the Broad Institute website. This package contains sets of genes found by individual experiments to be associated with specific phenotypes. The complete GSEA results for all utilized gene sets in this and all subsequent GSEAs are included as Supporting Information (Tables S4, S5, S6, S7, S8, S9, S10, S11, S12). Consistent with our observation of individual gene expression, the GSEA performed using curated gene sets showed that in forskolin treated basilar papillae many gene sets associated with cell cycle control are significantly enriched at various time points. For example, at 24, 48, and 72 hours there is enrichment of the following gene sets: SERUM_FIBROBLAST_CELLCYCLE [58], HSA04110_CELL_CYCLE [59], [60], [61], CELL_CYCLE [62], CELL_CYCLE_KEGG [59], [60], [61], GOLDRATH_CELLCYCLE [63], CELL_CYCLE_ CHECKPOINT, G1_TO_S_CELL_CYCLE_REACTOME [64], and BRENTANI_CELL_CYCLE [65] in explants exposed to forskolin (Table 4). The enrichment statistics for all gene sets used in this GSEA are included as Supporting Information (Tables S4, S5, S6). These findings are expected and instill confidence about the rest of the enrichment analysis results, given that forskolin treatment is known to stimulate S-phase entry in normally quiescent cells [22], [23].

**Table 4:** Curated gene set enrichment analysis results.

Gene Set	Enriched at 24H	24H P-Value	24H FDR	Enriched at 48H	48H P-Value	48H FDR	Enriched at 72H	72H P-Value	72H FDR
SERUM_FIBROBLAST_CELLCYCLE	*Forskolin*	*0.012*	*0.053*	*Forskolin*	*<0.001*	*<0.001*	*Forskolin*	*<0.001*	*<0.001*
HSA04110_CELL_CYCLE	*Forskolin*	*0.002*	*0.014*	*Forskolin*	*<0.001*	*<0.001*	*Forskolin*	*<0.001*	*<0.001*
CELL_CYCLE	*Forskolin*	*<0.001*	*0.004*	*Forskolin*	*<0.001*	*<0.001*	*Forskolin*	*<0.001*	*<0.001*
CELL_CYCLE_KEGG	*Forskolin*	*<0.001*	*0.004*	*Forskolin*	*<0.001*	*<0.001*	*Forskolin*	*<0.001*	*<0.001*
GOLDRATH_CELLCYCLE	*Forskolin*	*0.018*	*0.061*	*Forskolin*	*<0.001*	*<0.001*	*Forskolin*	*<0.001*	*0.002*
CELL_CYCLE_CHECKPOINT	*Forskolin*	*<0.001*	*0.003*	*Forskolin*	*0.002*	*0.008*	*Forskolin*	*<0.001*	*0.003*
G1_TO_S_CELL_CYCLE_REACTOME	*Forskolin*	*<0.001*	*0.002*	*Forskolin*	*0.006*	*0.023*	*Forskolin*	*0.013*	*0.054*
BRENTANI_CELL_CYCLE	*Forskolin*	*<0.001*	*0.004*	*Forskolin*	*<0.001*	*<0.001*	*Forskolin*	*0.018*	*0.059*
HSA04330_NOTCH_SIGNALING_PATHWAY	Forskolin	0.577	0.679	*Forskolin*	*0.037*	*0.084*	Forskolin	0.896	0.949
STEMCELL_COMMON_UP	*Forskolin*	*<0.001*	*0.001*	*Forskolin*	*<0.001*	*<0.001*	*Forskolin*	*<0.001*	*<0.001*
STEMCELL_COMMON_DN	Control	0.107	0.294	*Control*	*<0.001*	*0.022*	*Control*	*<0.001*	*0.008*
REN_E2F1_TARGETS	Forskolin	0.141	0.278	*Forskolin*	*0.002*	*0.007*	*Forskolin*	*<0.001*	*0.001*
VERNELL_PRB_CLSTR1	*Forskolin*	*0.006*	*0.054*	*Forskolin*	*<0.001*	*<0.001*	*Forskolin*	*<0.001*	*<0.001*
BRENTANI_DEATH	*Forskolin*	*<0.001*	*0.004*	*Forskolin*	*0.039*	*0.094*	Forskolin	0.853	0.914
HSA04210_APOPTOSIS	*Forskolin*	*<0.001*	*0.017*	*Forskolin*	*0.004*	*0.012*	*Forskolin*	*0.016*	*0.049*
APOPTOSIS	*Forskolin*	*0.002*	*0.010*	*Forskolin*	*0.004*	*0.044*	Control	0.308	0.454
APOPTOSIS_GENMAPP	*Forskolin*	*0.016*	*0.052*	*Forskolin*	*0.002*	*0.019*	Forskolin	0.146	0.256
PASSERINI_APOPTOSIS	Forskolin	0.085	0.207	*Forskolin*	*0.015*	*0.034*	*Control*	*0.045*	*0.132*

Shown are the curated gene set enrichment analysis results for selected gene sets of interest at 24, 48, and 72 hours. The gene sets shown here were downloaded from the BROAD Institute website and represent empirically defined sets of genes. References for each gene set are provided in the text. Italicized are those gene sets with p-value <0.05 and false discovery rate (FDR) <0.25, which are taken as significant. Gene sets are directly as they appear in the Molecular Signatures Database on the Broad Institute website.

The GSEA based on the curated gene sets also revealed that targets of E2F1, a transcription factor known to be important for cell cycle control, in human primary fibroblast cells identified by ChIP analysis (REN_E2F1_TARGETS [66]) are enriched in the proliferating auditory epithelia at both 48 and 72 hours. Further, a set of genes in the retinoblastoma pathway identified by microarray analysis in human cell lines (VERNELL_PRB_CLSTR1 [67]) was found to be significantly enriched after 24, 48, and 72 hours in culture with forskolin. These findings further underscore the potentially important role of the E2F1 transcription factor and the retinoblastoma protein pathway in auditory hair cell regeneration.

A particularly interesting result of the GSEA using the curated gene sets is the observation that a set of genes found to be upregulated in mouse embryonic, neural and hematopoietic stem cells (HSC) relative to differentiated brain and bone marrow cells (STEMCELL_COMMON_UP [68]) is also enriched in the basilar papilla after culture with forskolin for 24, 48, or 72 hours. Further, a set of genes found to be downregulated in stem cells (STEMCELL_COMMON_DN [68]) in the same study were also enriched in the quiescent tissue relative to proliferating tissue after 48 or 72 hours in culture, suggesting that genes downregulated in stem cells are also downregulated in the proliferating basilar papilla.

Though not widely accepted, there is evidence for the existence of stem cells within the postnatal organ of Corti [69]. Additionally, approximately 4% of supporting cells show stem cell-like behavior in that they undergo multiple rounds of division [70]. Recent work has shown that murine embryonic and induced pluripotent stem cells retain the capacity to differentiate into mechanosensitive cells which resemble immature hair cells [71]. It is interesting that genes found to be upregulated in HSCs specifically are also upregulated in proliferating basilar papillae given that HSCs are thought to remain in a state of quiescence until stimulated to proliferate by external signals [72], [73]. The chick basilar papilla is also quiescent until injury stimulates regeneration of hair cells by signals that have not yet been identified. It is therefore very interesting that proliferating basilar papillae overexpress genes that are also overexpressed in HSCs [68]. If the basilar papilla does in fact possess stem cells that share some properties with HSCs then it may be possible to stimulate the production of new hair cells using signals known to induce normally quiescent HSCs to proliferate. These results are consistent with the hypothesis that in forskolin treated basilar papillae some supporting cells are stimulated to dedifferentiate and express stem cell markers, prior to division and production of new hair cells.

It is worth noting that at 24 and 48 hours after exposure to forskolin, there is enrichment of the following curated gene sets, all of which are associated with cell death and/or apoptosis: BRENTANI_DEATH [65], HSA04210_APOPTOSIS [59], [60], [61], APOPTOSIS [64], APOPTOSIS_GENMAPP [64]. There is also enrichment of the gene set PASSERINI_APOPTOSIS [74] after 48 hours of exposure to forskolin. It is believed that forskolin induces proliferation in the inner ear without injury and subsequent regeneration, but this has never been definitively proven. However, the proliferation that is seen in the basilar papilla following exposure to forskolin is more robust than that seen following exposure to gentamicin, which causes hair cell death and regeneration [22]. Unpublished experiments performed for the same paper also did not show a qualitative loss of hair cells at 24, 48, or 72 hours, whereas hair cell ejection from the basilar papilla is seen as early as 30 hours following subcutaneous administration of gentamicin [75]. Markers of cell death in avian hair cells can appear even earlier following injury and begin with TIAR translocation as early as 12 hours after injury, before caspase activation which can occur as early as 30 hours after injury [76], [2]. Our gene expression data do not show differential expression of key markers of hair cell death such as caspase-3 and caspase-9 [76]. Regardless, the GSEA results suggest a possible contribution of apoptosis in forskolin induced proliferation in the avian inner ear. As the aim of the present study was to identify novel genes and pathways important for the production of new hair cells in deafened animals, the reported findings are of considerable interest regardless of a possible contribution of injury with subsequent regeneration in the forskolin-induced proliferation model of hair cell regeneration. Additional work will be needed to determine just how much, if any, of this proliferation is in fact the result of injury to the auditory epithelium.

### Mammalian gene set enrichment results

To determine which miRNAs were likely upregulated to give rise to the observed pattern of differential gene expression detected by microarray, an additional GSEA was performed using the mammalian gene sets which are based on genomic conservation between the human, rat, mouse, and dog genomes. These gene sets were used on our chicken gene expression data because they included not only transcription factor binding site, but also miRNA binding sites, which are fairly well conserved between species [42]. Using these mammalian gene sets and a false discovery rate cut-off of 0.25 as suggested by [40], 210 out of 783 gene sets were significantly enriched in the control condition and 37 in the forskolin condition at 72 hours (Table 1). As a negative control, enrichment of predicted targets of the transcription factor PAX6 was examined, given its known role in retinal development [77], [78] and absence of expression in the developing chicken inner ear [79]. That a pathway thought not to be important in the inner ear was not activated by forskolin exposure underscores that significantly enriched gene sets reflect real changes in gene expression (Supporting Information).

The difference in the number of gene sets associated with the control and forskolin conditions is striking, and it is worth pointing out that many of the gene sets associated with the control condition are miRNA gene sets, each of which represents a group of genes that are predicted targets of a specific miRNA. The association of these sets with the control condition implies that the miRNA target genes are down-regulated following forskolin treatment. Thus, we hypothesize that these miRNAs are upregulated in the forskolin group. We put forward this entire set of miRNAs as candidates whose role in hair cell regeneration and cellular proliferation warrants further investigation. Specific miRNAs and transcription factors of interest based on our analysis using the mammalian gene sets are presented in Table 5. A complete list of all significantly enriched gene sets from this analysis are included in the Supporting Information section (Tables S7, S8, S9).

**Table 5:** Mammalian gene set enrichment analysis results.

Gene Set	Enriched at 24H	24H P-Value	24H FDR	Enriched at 48H	48H P-Value	48H FDR	Enriched at 72H	72H P-Value	72H FDR
TGAATGT,MIR-181A,MIR-181B,MIR-181C,MIR-181D	Forskolin	0.475	1	*Forskolin*	*0.001*	*0.009*	*Control*	*<0.001*	*0.001*
ACTGTGA,MIR-27A,MIR-27B	Forskolin	0.238	1	Forskolin	0.406	1	*Control*	*0.002*	*0.042*
CAGTGTT,MIR-141,MIR-200A	Control	0.683	1	*Forskolin*	*0.017*	*0.648*	*Control*	*0.002*	*0.031*
V$E2F_Q6	Forskolin	0.033	0.273	*Forskolin*	*0.001*	*0.015*	*Forskolin*	*0.004*	*0.035*
V$E2F1_Q6	*Forskolin*	*0.003*	*0.009*	*Forskolin*	*0.003*	*0.102*	*Forskolin*	*<0.001*	*0.034*
V$E2F_Q4	*Forskolin*	*0.014*	*0.064*	*Forskolin*	*0.002*	*0.056*	*Forskolin*	*0.008*	*0.064*
V$E2F_Q4_01	*Forskolin*	*0.014*	*0.064*	*Forskolin*	*0.001*	*0.003*	*Forskolin*	*0.019*	*0.121*
V$E2F1_Q3	Forskolin	0.126	0.994	Forskolin	0.014	0.53	*Forskolin*	*0.006*	*0.121*
SGCGSSAAA_V$E2F1DP2_01	Forskolin	0.056	0.733	Forskolin	0.008	0.301	*Forskolin*	*0.014*	*0.121*
V$E2F1DP2_01	Forskolin	0.055	0.549	*Forskolin*	*0.001*	*0.016*	*Forskolin*	*0.031*	*0.14*
V$E2F1_Q4_01	Forskolin	0.254	1	*Forskolin*	*0.001*	*0.016*	*Forskolin*	*0.016*	*0.142*
V$E2F4DP1_01	Forskolin	0.055	0.677	*Forskolin*	*0.002*	*0.062*	*Forskolin*	*0.016*	*0.137*
V$E2F_02	Forskolin	0.056	0.601	*Forskolin*	*0.001*	*0.016*	*Forskolin*	*0.016*	*0.132*
V$E2F1DP1RB_01	Forskolin	0.033	0.35	*Forskolin*	*0.001*	*0.003*	*Forskolin*	*0.02*	*0.14*
V$E2F1DP1_01	Forskolin	0.055	0.771	*Forskolin*	*0.001*	*0.018*	*Forskolin*	*0.024*	*0.139*
V$E2F1_Q6_01	*Forskolin*	*0.003*	*0.009*	Forskolin	0.007	0.256	Forskolin	0.073	0.224
V$E2F_Q3	Forskolin	0.194	1	*Forskolin*	*0.002*	*0.069*	Forskolin	0.068	0.227

Shown are the mammalian gene set enrichment analysis results for selected gene sets of interest at 24, 48, and 72 hours. Italicized are those gene sets with p-value <0.05 and false discovery rate (FDR) <0.25, which are taken as significant. Gene sets are directly as they appear in the Molecular Signatures Database on the Broad Institute website.

Some of the miRNAs identified by enrichment analysis are interesting candidates for further study given what is already known about their function. For example, miR141 and miR200a are expressed in the chicken inner ear epithelium, and are important regulators of epithelial-mesenchymal transition [30], [80]. It is also interesting that the miR27 family showed up as one of the most significant gene sets in this particular analysis given the known role of this miRNA in cellular proliferation. Specifically, downregulation of this miRNA seems to decrease proliferation in the context of hepatic stellate cell activation, an early event in liver fibrosis [81]. MiR27a is overexpressed in human gastric adenocarcinoma, and inhibition of this miRNA limits proliferation [82]. Another study has also shown that the oncogenic activity of miR27a in human breast cancer cells is likely due to downregulation of genes controlling the G2-M cell-cycle checkpoint [83]. MiR181a is known to be expressed in the mammalian inner ear [28], and has a pro-proliferative role in human leukemia cells, which appears to be mediated by suppression of the cell cycle inhibitor p27 [33]. Given the abundance and nature of evidence for a role of miR181a, miR27a, and miR141/miR200a in cellular proliferation or inner ear development, we put these forward as particularly intriguing candidates for further study within the context of auditory hair cell regeneration.

### Chicken/frog gene set enrichment analysis

While the primary goal of the GSEA with the mammalian gene sets was to identify miRNAs that may be important for hair cell regeneration, interestingly some transcription factor gene sets were found to be up- and downregulated in the forskolin condition. This was surprising in light of the fact that these sets were based on portions conserved across only selected mammalian genomes, which were expected to differ significantly from gene sets based on the chicken genome. To verify the up- and downregulation of these gene sets in the chicken inner ear an additional GSEA was performed using gene sets based on scanning the portions of the chicken genome that are conserved with frog (*Xenopus tropicalis*) to look for enrichment of sets of genes sharing particular transcription factor recognition sites obtained from the TRANSFAC database. Using a q-value cutoff of 0.25 there were 41 gene sets associate with forskolin treatment, and 331 gene sets associated with forskolin treatment (see Table 1). The enrichment statistics for all gene sets included in this analysis are presented as Supporting Information (Tables S10, S11, S12). Of the six gene sets that were significantly enriched in the forskolin condition, four are sets of genes that share known E2F1 binding sites (Table 6). Additionally, some E2F1 gene sets are significantly enriched in forskolin treated tissue at 24 and 48 hours relative to untreated controls (Table 6), suggesting that upregulation of predicted E2F1 targets occurs early after forskolin treatment and is sustained until 72 hours in culture, when new cells are first seen [22]. Many E2F/E2F1 gene sets are also enriched in the forskolin treated tissue when the mammalian gene sets (Table 5) are used to compare the expression data from forskolin samples fixed after 24 and 72 hours in culture, suggesting agreement between the GSEAs performed using the mammalian gene sets and chicken/frog conservation gene sets. The identification of E2F/E2F1 by both analyses strongly suggests a role for this transcription factor in forskolin induced proliferation in the chicken inner ear.

**Table 6:** Conservation gene set enrichment analysis results.

Gene Set	Enriched at 24H	24H P-Value	24H FDR	Enriched at 48H	48H P-Value	48H FDR	Enriched at 72H	72H P-Value	72H FDR
V$E2F_Q2	*Forskolin*	*<0.001*	*<0.001*	*Forskolin*	*0.006*	*0.016*	*Forskolin*	*<0.001*	*0.003*
V$E2F1_Q3_01	*Forskolin*	*0.038*	*0.066*	*Forskolin*	*0.017*	*0.063*	Forskolin	0.024	0.415
V$E2F_03	*Forskolin*	*0.099*	*0.176*	*Forskolin*	*0.071*	*0.144*	*Forskolin*	*0.012*	*0.038*
V$E2F1DP1RB_01	Forskolin	0.793	0.840	Forskolin	0.905	0.933	*Forskolin*	*0.019*	*0.057*
V$E2F1_Q4_01	Forskolin	0.345	0.430	Forskolin	0.573	0.639	*Forskolin*	*0.041*	*0.096*
V$E2F_Q3	Forskolin	0.342	0.416	Forskolin	0.418	0.532	*Forskolin*	*0.038*	*0.101*
V$E2F_Q3_01	Forskolin	0.681	0.734	Forskolin	0.416	0.483	*Forskolin*	*0.038*	*0.108*
V$E2F_Q4	Forskolin	0.175	0.249	Forskolin	0.365	0.456	*Forskolin*	*0.040*	*0.108*
V$E2F1_Q6_01	Forskolin	0.422	0.477	Forskolin	0.245	0.332	Forskolin	0.058	0.115
V$E2F1_Q3	Forskolin	0.511	0.564	Control	0.306	0.437	*Forskolin*	*0.077*	*0.155*
V$E2F_Q6	Forskolin	0.964	0.965	Control	0.714	0.832	*Forskolin*	*0.073*	*0.155*
V$E2F1_Q6	Forskolin	0.330	0.395	Forskolin	0.245	0.332	*Forskolin*	*0.079*	*0.152*

Shown are the results of the conservation gene set enrichment analysis performed on gene expression data from 24, 48, and 72 hours in culture. These analyses were performed using gene sets based on transcription factor motifs in regions conserved between the chicken and frog genomes. The gene sets shown here were downloaded from the BROAD Institute website and represent empirically defined sets of genes. References for each gene set are provided in the text. Italicized are those gene sets with p-value <0.05 and false discovery rate (FDR) <0.25, which are taken as significant. Gene sets are directly as they appear in the Molecular Signatures Database on the Broad Institute website.

E2F1 is an intriguing candidate for further study given its known role in cell cycle control (reviewed in [84]) and potential role in inner ear development (reviewed in [85]). E2Fs bind hypophosphorylated pRBs which then sequester these factors. PRBs can be phosphorylated by cyclin-dependent kinases (CDKs), rendering these proteins unable to bind E2Fs thereby releasing these factors which then become transcriptionally active [86], [87], [88]. Interestingly, overexpression of E2F1 has been shown to induce quiescent REF-52 cells to enter S-phase [89]. E2F1 forms part of an ‘activating’ E2F complex that can interact with pocket proteins (pRBs) to control the cell cycle [90]. E2F1 exclusively binds the pRB RB1, and peaks in expression during the G1-S checkpoint [90]. Given the central role of E2Fs in cell cycle control, our finding that genes targeted by E2F1 are enriched among the genes that are overexpressed in the proliferating chick inner ear suggests a role for this transcription factor in hair cell regeneration.

### Functional microRNA181a studies

**Figure 3:**
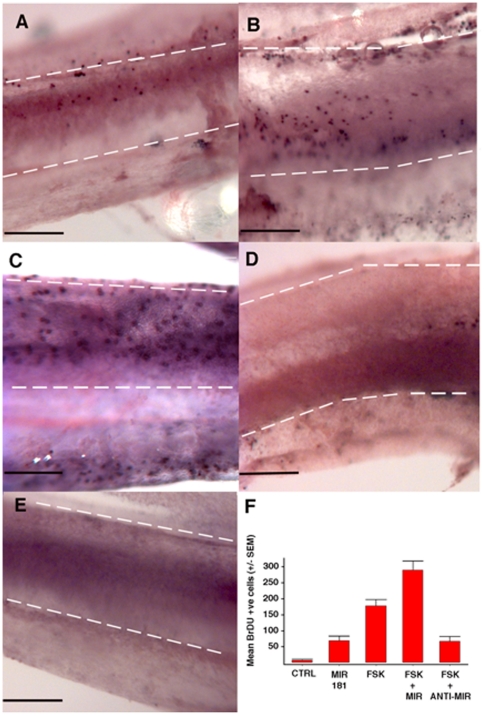
MicroRNA181a overexpression produces proliferation in the chicken basilar papilla. Basilar papillae from 0-day-old chicks were cultured for 72 hours in the presence of BrdU, the nuclear incorporation of which was detected by antibody labeling. In A–E sensory epithelia are outlined with dashed white lines. The neural edge of each epithelium is at the bottom, the abneural edge is at the top, the distal end is to the right and the proximal end is to the left of the image. BrdU-positive (i.e., recently divided) cells are immunohistochemically labeled and appear as purple dots. Individual cochleas were treated with 100 nM pre-miR181a (A), 100 µM forskolin (FSK) (B), FSK + 100 nM pre-miR181a (C), 100 µM FSK+ 100 nM anti-miR181a (D), or DMSO as a vector control (E). A pro-proliferative effect of miR181a can be appreciated. Further, knocking down endogenous miR181a appears to have a large suppressive effect on forskolin induced proliferation. Transfection was achieved in the first 24 hours of culture using X-tremeGENE siRNA Transfection Reagent in accordance with the manufacturer's instructions (Roche, Indianapolis, IN). The miRNA containing media was removed after 24 hours and replaced with normal medium. The summary of the average number of BrdU positive cells for the control (n = 7), miR181a (n = 8), forskolin (n = 8), forskolin + miR181a (n = 8), and forskolin + anti-miR181a (n = 3) conditions. The following comparisons were all statistically significant: control versus miR181a (p = 0.001), control versus forskolin (p<0.001), miR181a versus forskolin (p<0.001), forskolin versus forskolin plus anti-miR181a (p = 0.008) and forskolin versus forskolin plus miR181a (p = 0.005). Scale bars in panels A–E = 0.2 mm.

Of all the miRNAs whose predicted targets were significantly enriched in control relative to forskolin treated basilar papillae, miR181a was specifically selected for functional studies in light of its high level of statistical significance, known expression in the developing mammalian inner ear [28], and pro-proliferative role in human leukemia cells, which appears to be mediated by suppression of the cell cycle inhibitor p27 [33]. To test whether miR181a overexpression is sufficient to stimulate proliferation, chick basilar papilla explants were transfected with either pre-miR181a, which is converted to mature miR181a within cells, or a non-targeting negative control miRNA. Incorporation of the thymidine analog BrdU was used to assay proliferation. Basilar papillae transfected with pre-miRNA had on average 68.75 (+/− SEM = 13.54) BrdU positive cells, significantly more than organs transfected with a non-targeting miRNA which averaged only 7.00 (+/− SEM = 10.23) BrdU positive cells (p<0.01, Figure 3). MiR181a overexpression is therefore sufficient to stimulate proliferation in the normally quiescent chick auditory epithelium.

Basilar papillae cultured with forskolin for 72 hours and transfected with a non-targeting miRNA had on average 178.25 (+/− SEM = 19.02) BrdU positive cells, significantly more than is seen with miR181a overexpression (p<0.05) which suggests that miR181a upregulation may account for only a portion of forskolin's mitogenic effect (Figure 3). To determine whether miR181a expression is necessary for forskolin induced proliferation, some basilar papillae were cultured with forskolin for 24 hours, then transfected with anti-miR181a to suppress endogenous miR181a, then cultured with forskolin for an additional 24 hours. Basilar papillae transfected with only anti-miR181a without forskolin treatment had an average of only 14 (+/− SEM = 3.06) BrdU positive cells per epithelium, not significantly different from controls. Basilar papillae exposed to forskolin and transfected with anti-miR181a had an average of 67.33 (+/− SEM = 13.38) BrdU positive cells, significantly less than the basilar papillae that were cultured with forskolin and transfected with a negative control miRNA (p<0.01), suggesting that endogenous miR181a contributes to the pro-proliferative effect of forskolin in the chick inner ear.

To see whether miR181a could enhance the already robust pro-proliferative effect of forskolin, basilar papillae were transfected with pre-miR181a and cultured in the presence of forskolin for 72 hours. These basilar papillae averaged 289.63 (+/− SEM = 28.04) BrdU positive cells per basilar papilla, which is significantly higher than the forskolin effect alone (p<0.01). This observation reaffirms the finding that endogenous miR181a is an important mediator of forskolin induced proliferation in the chick inner ear and is limited in part by the ability of forskolin to stimulate upregulation of miR181a.

**Figure 4:**
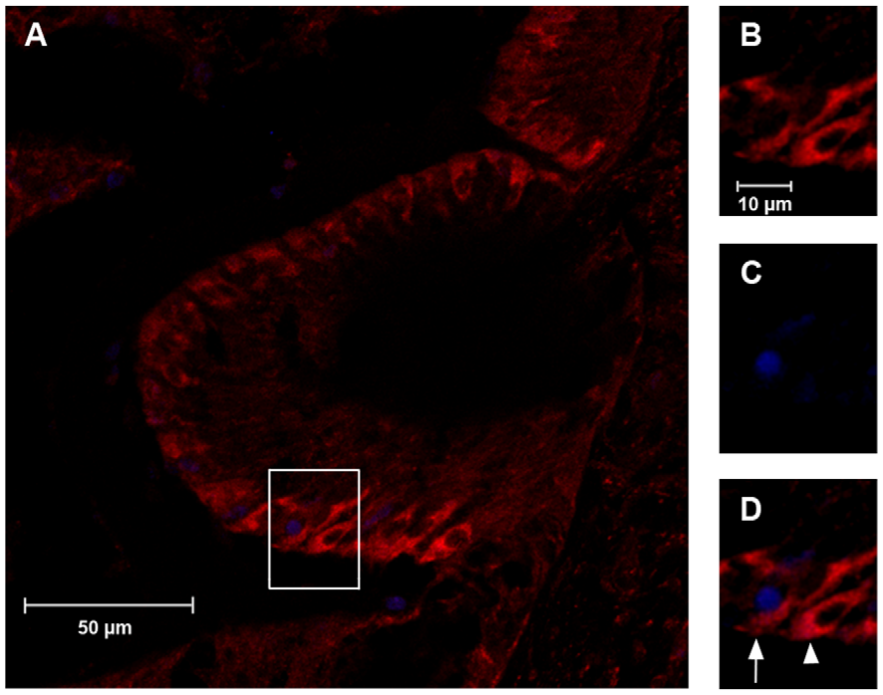
Some new cells produced by miR181a transfection express the early hair cell marker myosin VI. Shown is a confocal image of a basilar papillae that was transfected with pre-miR181a, cultured for 72 hours, then cryosectioned and labeled for BrdU (blue) and the early hair cell marker myosin VI (red) (**A**). The apical surface of hair cells is to the left of the image and the neural edge of the basilar papillae is toward the bottom. The tectorial membrane has been removed to allow for antibody labeling. The inset of **A** is also shown in panels **B** (myosin VI), **C** (BrdU) and **D** (merge). The arrow in **D** shows a cell that expresss myosin VI and has a BrdU positive nucleus, whereas the arrowhead shows a cell that is labeling for myosin VI but not BrdU. The double-labeled cell is one that has been stimulated to divide and subsequently begin differentiating toward a hair cell phenotype. The scale bar in **B** also applies to **C** and **D**.

Given the observation that miR181a overexpression is sufficient to stimulate proliferation in the chick auditory epithelium, basilar papillae were transfected with pre-miR181a or nontargeting negative control miRNA and then cryosectioned and labeled for BrdU and the early myosin VI to determine whether any of the newly produced BrdU positive cells express the early hair cell marker. As is seen in Figure 4, some cells labeling for BrdU 72 hours after transfection with pre-miR181a are located at the most apical portion of the basilar papilla, an area occupied by only hair cells and thin supporting cell processes. Further, some cells with nuclear BrdU labeling also have cytoplasmic myosin VI labeling. No such co-labeling cells were observed in basilar papillae transfected with a non-targeting miRNA. These results suggest that miR181a overexpression results in the production of new hair cells.

It is not surprising that a single miRNA is able to stimulate S-phase entry in the avian auditory epithelium given the mounting evidence that miRNAs can affect cell cycle regulation in a variety of systems. MiR181a specifically has been shown to have a pro-proliferative effect in human myeloid leukemia cells [33]. Interestingly, this effect appears to be mediated in part by producing downregulation of p27, which may represent a barrier to hair cell regeneration in the mammalian cochlea [34], [35], [91], [92]. There is also evidence that many different miRNAs, including miR181a, are expressed in the developing mouse inner ear [28]. Despite these findings, studies on the role of miRNA in hair cell regeneration are limited in number and scope. One study revealed that the let-7 family of miRNA is downregulated in the regenerating newt auditory epithelium [32]. An interesting translational correlate to this finding is that let-7 reduces tumor growth in a rodent model of lung cancer, providing additional evidence for the antiproliferative effects of this particular family of miRNA [93]. Studies such as these underscore the importance of examining more closely the role of miRNA in hair cell regeneration, as induced underexpression of antiproliferative genes may ultimately allow regeneration of hair cells in mammals.

In summary, we have identified a possible role for miR181a in avian auditory hair cell regeneration. We have further identified additional miRNAs and transcription factors as candidates that warrant further investigation as potential targets for therapeutics aimed at replacing lost hair cells. Presented additionally are results suggestive of the presence of stem cells with properties similar to HSCs in the regenerating chick inner ear. Of all the candidates identified miR181a is specifically put forward for further study in *in-vivo* mammalian models of induced hearing loss in light of its demonstrated proliferative effect in the chicken inner ear as well as the fact that a miRNA based therapeutic could potentially be locally delivered to the middle ear and passively absorbed through the round window. This scenario seems plausible given that small molecules such as gentamicin can be delivered to the inner ear of patients in this fashion [94]. Computational analyses of our gene expression data suggest that the transcription factor E2F1 may also play a role in hair cell regeneration when many normally quiescent supporting cells are stimulated to re-enter the cell cycle and divide prior to differentiating into new hair cells. Additional functional studies will be necessary to ultimately determine what ability, if any, these players have to replace lost hair cells and result in recovery of compromised hearing thresholds in mammals.

## Supporting Information

Table S1 Genes differentially expressed (p<0.05, fold-change >2) between control and forskolin treated basilar papillae after 24 hours in culture.
(0.02 MB XLS)

Table S2 Genes differentially expressed (p<0.05, fold-change >2) between control and forskolin treated basilar papillae after 48 hours in culture.
(0.03 MB XLS)

Table S3 Genes differentially expressed (p<0.05, fold-change >2) between control and forskolin treated basilar papillae after 72 hours in culture.
(0.52 MB XLS)

Table S4 Gene set enrichment analysis statistics. Curated gene sets enriched in the control and forskolin samples after 24 hours.
(0.25 MB XLS)

Table S5 Gene set enrichment analysis statistics. Curated gene sets enriched in the control and forskolin samples after 48 hours.
(0.25 MB XLS)

Table S6 Gene set enrichment analysis statistics. Curated gene sets enriched in the control and forskolin samples after 72 hours.
(0.25 MB XLS)

Table S7 Gene set enrichment analysis statistics. Mammalian gene sets enriched in the control and forskolin samples after 24 hours.
(0.19 MB XLS)

Table S8 Gene set enrichment analysis statistics. Mammalian gene sets enriched in the control and forskolin samples after 48 hours.
(0.19 MB XLS)

Table S9 Gene set enrichment analysis statistics. Mammalian gene sets enriched in the control and forskolin samples after 72 hours.
(0.20 MB XLS)

Table S10 Gene set enrichment analysis statistics. Chicken/frog conservation gene sets enriched in the control and forskolin samples after 24 hours.
(0.15 MB XLS)

Table S11 Gene set enrichment analysis statistics. Chicken/frog conservation gene sets enriched in the control and forskolin samples after 48 hours.
(0.15 MB XLS)

Table S12 Gene set enrichment analysis statistics. Chicken/frog conservation gene sets enriched in the control and forskolin samples after 72 hours.
(0.15 MB XLS)
